# Giant Tug Lesion of the Soleus

**DOI:** 10.5334/jbsr.2381

**Published:** 2021-02-15

**Authors:** Dima Al Jahed, Filip Vanhoenacker

**Affiliations:** 1 AZ Sint-Maarten, Mechelen/Faculty of Medicine and Pharmacy, University of Brussels, Brussels, BE; 2 AZ Sint-Maarten and University (Hospital) Antwerp/Ghent, BE

**Keywords:** tug lesion, magnetic resonance imaging, computed tomography

## Abstract

**Teaching point:** A giant soleus tug lesion is a benign pseudo-tumoral excrescence at the attachment of the soleus muscle at the posterior upper third of the tibia.

## Case Presentation

**Figure 1:**
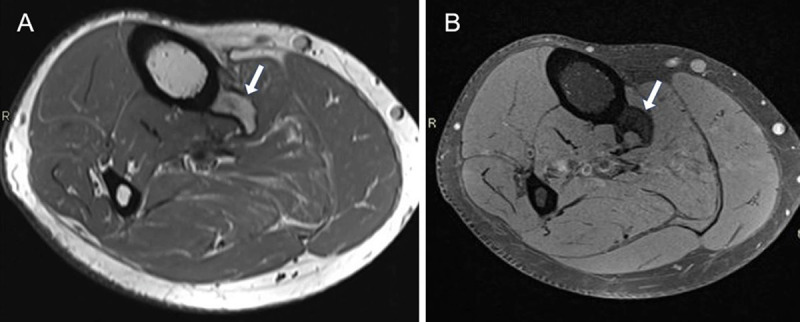


A 64-year-old man presented with intermittent pain during flexion of the foot and swelling on the medial side of the right lower leg. Magnetic resonance imaging (MRI) showed an osseous structure at the posterior margin of the proximal tibial shaft (***Figure 1A***, T2-WI, arrow). The lesion contained normal bone marrow (***Figure 1B***, FS T1-WI, arrow).

**Figure 2:**
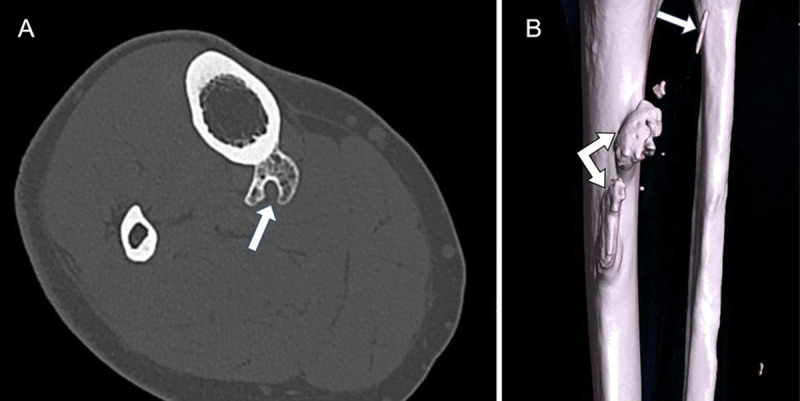


Subsequent CT examination confirmed the presence of a bony excrescence at the posteromedial tibial diaphysis with bifid morphology (***Figure 2A***, axial). A 3D reconstruction showed that the lesion was predominantly located on the course of the attachment of the soleus muscle at the tibia with an additional smaller component at the fibular attachment of the soleus (***Figure 2B***, posterior view, arrows). The diagnosis of a giant tug lesion of the soleus was made.

## Discussion

A tug lesion is a benign excrescence at the insertion of tendons or skeletal muscles at the adjacent bone. A tug lesion at the attachment of the soleus muscle at the tibia and fibula is a characteristic example [1]. Patients may be asymptomatic or present with pain due to local friction on the calf muscles during foot flexion.

Radiographically, a cortical irregularity or reactive bone formation, sclerosis, or a combination of these is seen. Small lesions are often difficult to detect on conventional radiographs.

Giant tug lesions are rare and may mimic other benign cortically based lesions, including myositis ossificans and cartilaginous exostosis or malignant lesions such as juxtacortical osteosarcoma. In myositis ossificans, there is often a recent clinical history of a trauma, and the lesion originates in the muscles with progressive peripheral calcification and ossification, which may fuse with the underlying cortex in the mature stage.

A cartilaginous exostosis can be distinguished from a tug lesion on MRI by the presence of a cartilage cap and the continuity of medullary and cortical bone of the lesion and that of the host bone. In our case, there was no cartilage cap on T2-WI and the cortex of the tibia was clearly separated from the bony excrescence.

Juxtacortical osteosarcomas have a predilection for the posterior cortex of the distal femur and are not restricted to the soleus attachment.

CT is very useful for lesion characterisation. The clue to the diagnosis is its location along the posterior medial margin of the proximal tibial shaft and at the fibular attachment of the soleus. 3D reconstructions may be very helpful to demonstrate the lesion on the course of its muscular attachment. This will avoid unnecessary invasive diagnostic tests, such as biopsy.
